# Using knowledge translation to establish a model of hospital-based early supported community reintegration for stroke patients in South Korea

**DOI:** 10.1186/s12913-021-07400-5

**Published:** 2021-12-20

**Authors:** Eunjoo Kim, Minyoung Lee, Eun-Hye Kim, Hyoung Jun Kim, Mijung Koo, In Yae Cheong, Hyun Choi

**Affiliations:** 1 Department of Rehabilitation Medicine, National Rehabilitation Center, Seoul, South Korea; 2 Department of Healthcare and Public Health Research, National Rehabilitation Research Institute, Seoul, South Korea; 3 Department of Clinical Research for Rehabilitation, National Rehabilitation Research institute, Seoul, South Korea

**Keywords:** Community reintegration, Return home, Knowledge translation, Knowledge-to-action cycle, Patients with stroke

## Abstract

**Background:**

In 2019, the South Korean government started designating rehabilitation medical institutions to facilitate the early return of patients with stroke (PWS) to their communities after discharge. However, a detailed operating model has not yet been suggested. We aimed to develop a hospital-based early supported community reintegration model for PWS that is suitable for South Korea based on knowledge translation in cooperation with clinical experts and PWS.

**Methods:**

Clinical experts (*n* = 13) and PWS (*n* = 20) collaboratively participated in the process of developing the early supported community reintegration model at a national hospital in South Korea, using the following phases of the knowledge-to-action cycle: (1) identifying knowledge, (2) adapting the knowledge to the local situation, (3) assessing barriers and facilitators to local use of knowledge, and (4) tailoring and developing the program. Barriers and facilitators to local use of knowledge were assessed multidimensionally at the individual, interpersonal, organizational, and community level based on the social-ecological model. Literature reviews, workshops, individual and group interviews, and group meetings using nominal group technique were conducted in each phase of the knowledge-to-action cycle.

**Results:**

Each phase of the knowledge-to-action cycle for developing the early supported community reintegration model and a newly developed model including the following components were reported: (1) revision of strategies of organizations related to community reintegration support, (2) establishment of a multidepartmental and multidisciplinary community reintegration support system, (3) standardization of patient-centered multidisciplinary goal setting, (4) multidimensional classification of community reintegration support areas, and (5) development of guidelines for a tailored community reintegration support program.

**Conclusions:**

We designed a hospital-based multidimensional and multidisciplinary early supported community reintegration model that comprehensively included several elements of community rehabilitation in connection with hospitals and communities, taking into account the South Korean situation of lacking community rehabilitation infrastructure. In developing a guideline for a tailored community reintegration support program, we attempted to take into consideration various situations faced by PWS in South Korea, which is in a transitional stage for community rehabilitation. It is expected that this early supported community reintegration model can be referenced in other countries that are in a transitional stage of community rehabilitation.

## Background

South Korea is expected to face an ultra-aged society in which more than 20% of the entire population will be 65 years or older by 2025. Along with the steep aging rate, the population of patients with stroke (PWS) is also growing, making stroke the fourth leading cause of death in South Korea and causing the third highest medical cost among all types of disability [1, 2].

In South Korea, patients often had to be discharged from hospital without receiving sufficient rehabilitation to return to their communities because the length of hospital stay covered by the national insurance was limited, i.e., 100% covered for up to 15 days, 90% for 16–30 days, and 85% afterward [3]. To make matters worse, the infrastructure for rehabilitation in the community was not well-equipped, eventually forcing patients to be readmitted to another hospital after discharge [4]. Thus, reform of the medical system in South Korea is desperately needed to support the early discharge of the patients through intensive rehabilitation during the hospitalization and ultimately support early return to their communities.

In North America and Europe, PWS have traditionally received a substantial part of their rehabilitation after the acute phase in an inpatient rehabilitation facility [5, 6], while to reduce the length of hospital stay, the late 1990s saw a move toward more innovative models of care, known as the early supported discharge, in which PWS were discharged earlier and received rehabilitation and support within their home environments [7, 8]. In Japan, the ‘Kaifukuki rehabilitation ward’ system where intensive and inpatient rehabilitation were carried out was introduced in the year 2000 as part of a national effort to reduce the length of hospital stay as the elderly population increases [9–13]. Patients who still need assistance in activities of daily living after treatment of specific disabling diseases in acute care hospitals are transferred to a Kaifukuki rehabilitation ward [9–13] which is comparable to the inpatient rehabilitation facility in North America and Europe.

The South Korean government has designated some hospitals as rehabilitation medical institutions in 2019 to provide hospital-based intensive and personalized rehabilitation to facilitate the early return of patients to the community, benchmarking the inpatient rehabilitation facility and Kaifukuki rehabilitation ward systems. However, a detailed operating model for rehabilitation medical institutions has not been suggested yet. Previously developed models, such as inpatient rehabilitation facility, early supported discharge, and Kaifukuki rehabilitation ward, have the common purpose of encouraging patients to leave the hospital ‘early’ through the provision of intensive rehabilitation, so these models can be referred to in the development of an operating model for rehabilitation medical institutions. However, there are differences in national policies, medical systems, and community situations among countries, and accordingly, the barriers experienced by South Korean clinical experts and patients might differ from those in other countries. Thus, it is necessary to develop an operating model for rehabilitation medical institutions, taking into account the situation in South Korea.

In this study, we adopted knowledge translation to develop a hospital-based early supported community reintegration model that is suitable for the situation in South Korea because knowledge translation focuses on closing existing gaps between available prior knowledge and real-world demands [14]. In the knowledge translation process of this study, both clinical experts and PWS collaboratively participated in improving the acceptability and sustainability of the model.

## Methods

### Theoretical framework and study design

The knowledge translation method was defined by the Canadian Institutes of Health Research as ‘a dynamic and iterative process that includes the synthesis, dissemination, exchange, and ethically sound application of knowledge within a complex system of interactions among researchers and community partners [15]. Essentially, knowledge translation focuses on closing the gaps that exist between what is known from the knowledge and what is carried out in practice [14, 15]. Graham et al. [14] developed a conceptual framework for systematic translation from knowledge products to actions through several phases, entitled ‘knowledge-to-action cycle’. The knowledge-to-action cycle entails: (1) identifying knowledge; (2) adapting the knowledge to the local situation; (3) assessing barriers and facilitators to its use in practice; (4) selecting, tailoring, and using strategies to implement the use of this knowledge; (5) monitoring knowledge use; (6) evaluating outcomes; and (7) sustaining the use of knowledge over time. In the field of medicine, knowledge translation has been recently widely used to analyze the experiences and needs of patients and caregivers [16, 17], to develop interventions and guidelines [18], and to verify the effectiveness of newly developed interventions and guidelines [19].

In this study, we reported the key process of development of a hospital-based early supported community reintegration model applying the framework of knowledge-to-action cycle. As a method of reaching consensus on the opinions of researchers and clinical experts in the process of developing the model, the nominal group technique, which combined quantitative and qualitative data collection and analysis, was adopted [20, 21]. The nominal group technique is useful tool for facilitate groups in ideas generation, decision-making and priority setting. In the phase of assessing barriers and facilitators to local use of knowledge, qualitative research method was employed in order to explore the clinical experts and patients perceived experience.

### Study setting

This study was conducted in a national hospital in Seoul, which had been designated as a rehabilitation medical institution in 2019. Between 2012, and before the hospital received the rehabilitation medical institution designation, a community reintegration support team comprised solely of social workers had been established to provide support for patients returning to the community in addition to rehabilitation. This community reintegration support team provided several services to support the community integration of patients, and patients were able to participate in services of interest at their own choice during the hospitalization period for up to 90 days due to limitations of the national insurance system. After being designated as a rehabilitation medical institution, it became necessary to develop an operating model to support the patients’ community reintegration during hospitalization periods of up to 180 days.

### Research partners

#### Clinical experts in all phases of knowledge-to-action cycle

A 1-year project team of researchers and clinical experts was formed in the hospital to develop an early supported community reintegration model. A total of 13 multidisciplinary clinical experts, including a medical doctor (*n* = 1), nurses (*n* = 2), physical (*n* = 2) and occupational therapists (*n* = 2), a psychological counselor (*n* = 1), and social workers (*n* = 5), participated in this study, all roles related to community reintegration support in the hospital. Of those, 11 were female, and their clinical experience ranged from 7 to 30 years. The researchers sought to establish equitable partnerships with clinical experts during the entire research process.

#### PWS in the phase of assessing barriers and facilitators to local use of knowledge

Purposive and snowballing sampling strategies were employed to identify patient partners [22]. Clinical experts in partnership with researchers recommended patients from among those (1) who had been hospitalized for more than 2 months or had been discharged for less than a year, (2) who had been provided with community reintegration support services by the community reintegration support team, and (3) who had normal cognition ability sufficient to conduct individual interviews. It was planned that the number of PWS would be less than or equal to 20 to facilitate the researcher’s close association with the respondents, and enhance the validity of fine-grained, in-depth inquiry [23]. A total of 20 patients participated in this study. Four of them were female, and their age ranged from 24 to 73 years. The level of physical function varied from Modified Barthel Index 30 to 93 points. Approval for this study was obtained from the National Rehabilitation Center (NRC-2018-02-009). Written informed consent was obtained from all clinical experts and PWS.

### Procedure of development of a hospital-based early supported community reintegration model: application of the knowledge-to-action cycle

The research team judged that the community reintegration support program previously developed by the community reintegration support team has limitations in practically realizing early community reintegration, the purpose of a rehabilitation medical institution, and that the existing model was not suitable in a situation where the national insurance coverage for a rehabilitation medical institution has increased to a maximum of 180 days. Accordingly, the research team reached an agreement on the necessity of expanding and updating the existing community reintegration support services at the team level to a multidepartmental, multidisciplinary model at the organizational level, which could cover hospitalization periods of up to 180 days. Researchers and clinical experts regularly met every other week for 12 weeks to design the early supported community reintegration model for PWS through knowledge-to-action cycle phases as follows [14]:

#### Phase 1. Identifying knowledge

As a reference for developing a new model, the researchers investigated through literature reviews the support systems for community reintegration in the UK and Japan, which faced an aging society earlier than South Korea. Specifically, researchers investigated the definition, purpose, target audience, operational strategies, and effectiveness of early supported discharge and Kaifukuki rehabilitation ward. All research teams shared the results of their literature reviews through seminars.

#### Phase 2. Adapting the knowledge to the local situation

Researchers and clinical experts co-learned each other’s knowledge to adapt the identified information to the local situation. One researcher with experience in conducting research using the knowledge translation method presided over a workshop on the knowledge-to-action cycle framework so that all research teams could understand each phase of the knowledge-to-action cycle and develop their ability to adapt knowledge in the literature to real-life situations. Similarly, social workers explained the services which had been provided by the community reintegration support team. The research team discussed which elements from previous studies should be referenced to update the current model using the nominal group technique.

#### Phase 3. Assessing barriers and facilitators to local use of knowledge

The research team attempted to assess the barriers and facilitators recognized by the PWS and clinical experts engaged in previously developed community reintegration support services using individual interview for the PWS and group interview for clinical experts, thereby identifying additional points that should be taken into account in future model developments. Clinical experts gave opinions on their experience that environmental, as well as personal, factors would influence the experience of clinical experts and PWS interactively while participating in community reintegration support services; therefore, the researchers proposed to assess barriers and facilitators multidimensionally based on the socio-ecological model [24, 25]. The socio-ecological model suggests a comprehensive approach that integrates multiple levels of influence on impact behavior, including individual, interpersonal, organizational, and community factors [26, 27]. In the field of public health, the socio-ecological model has been used to identify barriers and facilitators in a multidimensional manner [28, 29] and to develop interventions that reflect identified multidimensional factors [30], although it rarely has been applied to hospital-based studies.

#### Phase 4. Tailoring and developing the program

The research team used the nominal group technique in group meetings to discuss and determined the components of the early supported community reintegration model and detailed content of each component. In the process of developing the model, the research team comprehensively considered identified knowledge and barriers and facilitators, as well as organizational structure, resources, and community situation.

### Data collection and analysis

The nominal group technique was adopted for reaching consensus on the opinions of researchers and clinical experts in adapting the knowledge to the local situation and developing the model [20, 21]. The nominal group technique uses a highly structured meeting for ideas generation, decision-making and priority setting about a given issue. It consists of two rounds in which panellists rate, discuss, and then rerate a series of items or questions. In this study, the nominal group technique was facilitated by one researcher on the topics about how to adapt the knowledge to the local situation and develop the model. The nominal group technique was structured as follows: (1) researchers and clinical experts spent several minutes writing down their opinions about the topic in question; (2) each, in turn, suggested one idea to the facilitator, who recorded it on a flip chart; (3) similar suggestions were grouped together, where appropriate. There was a group discussion to clarify and evaluate each idea; (4) each privately ranked each idea (round 1); (5) the ranking was tabulated and presented; the overall ranking was discussed and reranked (round 2).

In assessing barriers and facilitators to local use of knowledge, individual interviews were conducted for the PWS considering the patients’ convenience regarding time and place. Individual interviews were semi-structured to help patients answer open-ended questions, as follows: (1) What do you think motivated or facilitated your engagement in the community reintegration support program? (2) What do you think were the barriers to your engagement in the community reintegration support program? Each interview took approximately 40–60 min to complete. For clinical experts, group interviews were conducted for each job group (i.e., the medical doctor, nurses, physical therapists, occupational therapists, and the psychological counselor). Group interviews were semi-structured as follows: (1) What do you think motivated or facilitated your support for community reintegration of patients? (2) What do you think were the barriers to your support for the community reintegration of patients? Each interview took approximately 60–90 min to complete. All interviews were conducted in the hospital and audio-recorded with the consent of the patients.

For qualitative analysis, all audio-recorded interviews were transcribed into text by two research assistants not participating in the study to avoid selective coding of information [31]. A summative content analysis was adopted to analyze the transcribed interviews by counting and comparing keywords in the text for the purpose of understanding the contextual meaning of the sentence [32]. One researcher and clinical expert independently coded the transcribed interviews by sentence and decided on the themes based on keywords that were presented in codes. Those themes were discussed among all the researchers and clinical experts to verify their accuracy and representativeness.

## Results

Thirteen clinical experts participated in all phases of the knowledge-to-action cycle, and twenty patients participated in assessing barriers and facilitators to local use of knowledge.

### Phase 1. Identifying knowledge

#### Identified knowledge 1: early supported discharge

Early supported discharge is a model that links inpatient care with community services and provision of intense rehabilitation services within the home environment at a level similar to the care provided in hospitals to enable appropriate stroke survivors to leave the hospital early, thereby reducing the length of hospital stay [7]. The purpose is to achieve maximum potential for independence in all aspects of stroke survivors’ and their caregivers’ lifestyles, acquiring the necessary skills to adapt to new situations [7]. According to the National Health Service of the UK, it is recommended to enter acute care at the hospital 3–7 days after stroke onset or initiate early supported discharge immediately after receiving hyperacute care up to 72 h after onset [33]. Previous studies suggested that early supported discharge services require 4–5 weeks on average to be effective [34].

Early supported discharge is strongly recommended for stroke patients with mild-to-moderate disability, where appropriate home-based coordinated stroke services are available [8]. Early supported discharge trials suggested that 15% of patients might be eligible based on objective measures of both physical and cognitive function, e.g., Barthel Index scores of 16–19 and a Mini-Mental State Examination score greater than 23, in addition to caregiver availability, suitability of the home environment, and proximity to the hospital [8]. Working-age PWS tend to have specific needs concerning their return to work, parenting, and psychosocial aspects of recovery [35]. Exclusion criteria were stroke survivors unwilling to participate in rehabilitation or where realistic achievable goals were not identified, as well as stroke survivors with severe symptoms [35].

To work effectively, early supported discharge services must have elements similar to those of stroke unit teams. Typical early supported discharge teams need to be multidisciplinary and have approximately 3.1 full-time equivalent staff (range 2.6–4.6) as follows: medical doctor (0.1), nurses (range 0–1.2), physiotherapists (1.0), occupational therapists (1.0), speech and language therapists (0.3), assistants (0.4), social workers (range 0–0.5), and secretarial support [35]. Access to therapy must be provided in a timely manner through agreed multidisciplinary goals to offer a holistic approach to rehabilitation.

In a Cochrane review [35], participants receiving early supported discharge services showed significant reductions in length of hospital stay equivalent to approximately 6 days. Early supported discharge also reduced the outcome of death or institutional care and extended activities of daily living scores and level of patient satisfaction, although there was no difference in the rate of readmission to hospital between early supported discharge and conventional services groups [35].

#### Identified knowledge 2: Kaifukuki rehabilitation ward

According to the medical service law of Japan, a Kaifukuki rehabilitation ward is ‘A ward for intensive rehabilitation based on a rehabilitation program co-created by physicians, nurses, physical therapists, and occupational therapists, to prevent a bedridden state and to promote home rehabilitation by improving the ability to perform activities of daily living in patients with cerebrovascular disease, hip fracture, and so on.’ [36] Thus, the main purposes of the Kaifukuki rehabilitation ward are improvement of activities of daily living, avoidance of prolonged bed rest, and accomplishment of home discharge [9–13]. Patients within 2 months after the onset of stroke are eligible for admission to a Kaifukuki rehabilitation ward, and the maximal length of hospital stay covered by the insurance is 150 days for stroke and 180 days for stroke combined with other neurological diseases of severe disability and cognitive disorders [9].

To be designated as a Kaifukuki rehabilitation ward, a multidisciplinary team is required to have a medical doctor (≥1), nursing staff (13–15 patients to 1 member of the nursing staff), nursing assistants (30 patients to 1 member of staff), physiotherapists (≥2), occupational therapists (≥1), speech and language therapists (optional), social workers (optional), and nutritionists (optional) [36]. To facilitate an interdisciplinary team approach, the Kaifukuki rehabilitation ward team must provide patients and their families with a comprehensive monthly rehabilitation plan, including information about planned goals, achieved goals, rehabilitative approaches to achieve the remaining goals, discharge planning, and social resources necessary for home discharge [9–13].

The Annual Survey Committee of the Kaifukuki rehabilitation ward Association of Japan examined the achievements of the Kaifukuki rehabilitation ward system 10 years after its introduction [13]. As a result, the mean age increased gradually, and the number of PWS in Kaifukuki rehabilitation wards steadily increased. This gradual increase is supposedly related to the progressive aging of the Japanese population. The rate of home discharge increased. In 2012, 68% of PWS were discharged home. However, the length of hospital stay in Kaifukuki rehabilitation wards was not reduced in these 10 years [13]. The unchanged length of hospital stay in Kaifukuki rehabilitation wards might be secondary to the gradual increase in severely disabled inpatients, who had been included as target subjects of Kaifukuki rehabilitation wards according to Japanese health policy changes.

### Phase 2. Adapting the identified knowledge to the local situation

Previously developed community reintegration support services are largely classified into four programs and provided to applicants without specific eligibility criteria: (1) initial counseling, including personal history taking and social work consultation within 2 weeks after admission; (2) basic community reintegration support program including recreational activities, such as painting and singing, and social life experience, such as watching movies and taking a walk in groups; (3) advanced community reintegration support programs including social life training, such as using public transportation and facilities in the community, mentoring, and vocational rehabilitation; (4) discharge preparation programs, including smart home experience, house renovation, and community resources linkage 1 month before discharge.

To update the existing community reintegration support program, the research team agreed that the multidisciplinary approach was referenced both in the early supported discharge and Kaifukuki rehabilitation ward, such as organizing a multidisciplinary team, setting multidisciplinary goals, and sharing a comprehensive monthly rehabilitation plan, discharge plan, and social resources necessary after discharge through a multidisciplinary team conference. The research team also found that it was worth referring to measures for determining patients’ eligibility for early supported discharge and Kaifukuki rehabilitation ward, such as the onset of stroke, level of physical and cognitive function, caregiver availability, and suitability of the home environment.

### Phase 3. Assessing barriers and facilitators to local use of knowledge

What PWS and clinical experts felt as common barriers was identified primarily at the organizational and community level: ‘Absence of guidelines to provide a tailored community reintegration support program’ at the organizational level; and ‘Poor community infrastructure’ and as a consequence ‘Difficulties connecting patients to the community,’ as well as ‘The need for patients’ house renovation’ at the community level. In some cases, factors that PWS felt as barriers at the individual level affected the factors that clinical experts felt as barriers at the interpersonal level. For example, patients felt their ‘Weak perception of the necessity of returning to the community’ as a barrier to participating in the community reintegration support program, while clinical experts felt ‘Low priority of patients for participating in the community reintegration support program’ as a barrier to their provision of the community reintegration support program. Table 1 summarizes the barriers and facilitators at each level for both PWS and clinical experts.

**Table 1:** Identified barriers and facilitators based on the socio-ecological model

	People with stroke (***n*** = 20)	Clinical experts (***n*** = 13)
**Individual level**	•-Weak perception of the necessity of returning to the community •-Level of motor function •-Age	•-Lack of proficiency in work related to community reintegration support
**Interpersonal level**	•-Need for counseling for the patient’s family •-Need for counseling from peers	•-Need for standardization of work related to community reintegration support •-Patients’ low priority for participating in the community reintegration support program •-Need for psychological counseling for patients and their family
**Organization level**	•-Inadequate point of intervention for the community reintegration support program •-Need for education to improve one’s capabilities •-Absence of guidelines to provide a tailored community reintegration support program •-Lack of linkage between rehabilitation and community reintegration support programs	•-Lack of personnel •-Need for a multidisciplinary community reintegration support system •-Absence of guidelines to provide tailored a community reintegration support program •-Need to change the hospital policy from hospital-based rehabilitation to community reintegration support
**Community level**	•-Poor community infrastructure •-Need for house renovation	•-Difficulty connecting patients with the community •-Need for a renovation of the patient’s house

#### Individual level

PWS. (1) Weak perception of the necessity of returning to the community: In many cases, the purpose of a patient’s hospitalization was to ‘train arm and leg functions’ in order to return to the ‘previous state’ (before hospitalization), and many patients were planning to transfer to other hospitals after discharge to receive more rehabilitation. Therefore, patients often wanted to focus only on rehabilitation during the therapy rather than to participate in the community reintegration support program. (2) Level of motor function: Many patients felt burdened by participating in community reintegration support services such as cooking and painting in their current state of being unable to move the upper limb freely due to hemiplegia. (3) Age: Some young patients showed interest in vocational training and driving rehabilitation, whereas older patients generally did not feel the need for such training.

Clinical experts. (1) Lack of proficiency in work related to community reintegration support: When a physical therapist, who lacked experience in providing training in an external environment, accompanied a social life experience, it was sometimes not possible for them to provide tailored guides for patients to appropriately cope, for instance, with public transportation or walking on the road. If a prosthetic assistant explained assistive devices to patients, it was not always possible for these assistants to provide a tailored explanation because they were at times unable to accurately judge the status of each patient.

#### Interpersonal level

PWS. (1) Need for counseling for the patient’s family: PWS mentioned that family counseling was necessary both at the beginning of the hospitalization and before discharge because it was difficult not only for themselves but also for their families to accept the fact that they have become disabled and both of them did not know how to physically and mentally deal with situations they would face after discharge. (2) Need for counseling from peers: PWS mentioned the necessity of forming a mentor-mentee relationship among PWS in which they could easily ask how they could live in each period 1, 5, and 10 years after discharge through fellow disabled people who had already returned to their communities.

Clinical experts. (1) Need for standardization of work related to community reintegration support: Clinical experts recognized the necessity of common standardized methods for goal setting and achievement evaluation among departments. (2) Patients’ low priority for participating in the community reintegration support program: Clinical experts felt that most of the patients were interested only in gait training, exercise, muscle strengthening, and cognitive function improvement during the hospitalization period, thinking that participation in the community reintegration support program was optional and that priority was not given to the community reintegration support program. (3) Need for psychological counseling for patients and their families: Some social workers and the clinical psychological counselor desperately felt the need for psychological counseling for patients and their families. Clinical psychological counselors suggested that psychological counseling should start from the beginning of the hospitalization because the symptoms are already rather advanced when patients feel the need for counseling.

#### Organizational level

PWS. (1) Inadequate point of intervention for the community reintegration support program: Regarding the time of recommendation for the community reintegration support program, the patients said that it would be better if the hospitalization period had passed about 1/3 to 2/3 because they thought that it was necessary to adjust to the hospital life and that the most urgent aim in the early stages of hospitalization was the ‘recovery of the body.’ (2) Need for education to improve one’s capabilities: PWS said that they were able and willing to take measures of their own once the therapist informed them of the state of their current health condition. As soon as they knew the extent of their disability and how to deal with it, they felt they would no longer require hospital care and would more likely return home. (3) Absence of guidelines to provide a tailored community reintegration support program: PWS recognized that clinical experts guided the community reintegration support program at their discretion without established decision criteria and provided the same program uniformly for all PWS. Some PWS argued that clinical experts should provide community reintegration support programs by grouping similar patients based on their level of motor function or age. (4) Lack of linkage between rehabilitation and community reintegration support programs: Many complaints addressed the lack of connection between rehabilitation and community reintegration support programs in coordinating contents and time. For example, PWS thought that if more therapists accompanied social life experiences, it would allow training PWS to walk or move correctly. PWS also complained that they often had an overlap between therapy and community reintegration support program times, and in such cases, they had to choose therapy, regretting that if the time had been adjusted in advance, they could have participated in both.

Clinical experts. (1) Lack of personnel: Clinical experts in all departments mentioned that there was a limit to the provision of community reintegration support services due to staffing problems. If nurses and therapists had participated in a home visit or social life experience, problems would have arisen preventing them from performing hospital-based treatments. Therefore, most activities had only been accompanied by a minimum of personnel. (2) Need for a multidisciplinary community reintegration support system: Clinical experts felt the need for cooperation between departments in providing community reintegration support services, which failed for various reasons, expressing regret that the quality of the service may eventually decline. For example, there was no established route to share common patient-oriented goals to be achieved during hospitalization or to formally exchange patient information between departments. It was also pointed out that there was no collaboration between departments in designing community reintegration support services such as smart homes, home visits, as well as social experiences and training. (3) Absence of guidelines to provide a tailored community reintegration support program: Since there were no decision criteria for determining which program to apply to which patient, the clinical experts provided the same program to all patients, whereas some experts modified the program based on their judgment of patients’ needs. (4) Need to change the hospital policy from hospital-based rehabilitation to community reintegration support: Clinical experts noted that rehabilitation and community reintegration support programs were currently separated, but rehabilitation integrated into the community reintegration support program needed to be carried out in spaces not limited to hospital wards.

#### Community level

PWS. (1) Poor community infrastructure: Patients were worried about the lack of infrastructure in the area where they had to return after discharge. The community health center where information could be provided was located far from their home, there were no suitable facilities for exercise or rehabilitation, or there were no facilities with parking lots, making it difficult to access the institution. As a result, patients found it more inconvenient to return home than to stay in the hospital, and this perception acted as an indirect barrier to prevent patient participation in a community reintegration support program. (2) Need for house renovation: Patients identified the housing environment they would return to as a problem. There were no grab bars in the bathroom, or the floor was tiled and, therefore, slippery.

Clinical experts. (1) Difficulty connecting patients with the community: Clinical experts complained that it was difficult to connect patients with their communities because of poor community infrastructure such as facilities and experts supporting community care of patients in the area where the patients would return. Therefore, patients often regarded that they were abandoned or gave up rehabilitation after they had been discharged from the hospital. (2) Need for a renovation of the patient’s house: Clinical experts said that even if there was infrastructure in the area where the patient would return, it would be difficult for them to return home unless problems concerning their living environment or means of transportation were resolved.

### Phase 4. Tailoring and developing the program

Through the nominal group technique, the research team determined the following five components of the early supported community reintegration model and detailed content of each component.

#### Revision of strategies of community reintegration support-related organizations

Based on the current research project, the institution’s strategic goal was revised from ‘providing hospital-based rehabilitation’ to ‘providing community reintegration support in connection with the hospital and community.’ The primary measures for the revised strategic goal were: (1) providing intensive individualized rehabilitation by a multidisciplinary professional team, (2) supporting patients to improve their physical activity voluntarily even after discharge, (3) providing driving rehabilitation to support the mobility rights of PWS, (4) establishing a system in connection with the community to continue follow-up management even after the patient’s discharge, (5) refurbishing the patient’s home environment according to the characteristics of the disability, and (6) reinforcing education to improve disability awareness.

#### Establishment of a multidepartmental and multidisciplinary community reintegration support system

It was decided to hold a total of three multidisciplinary conferences during the hospitalization period of up to 180 days, through which a multidisciplinary plan was established for each patient, and the progress of the patient’s condition was monitored. The first and second conferences were held within 60 and 120 days after stroke onset, and the third conference was held between 121 and 180 days. Physicians, nurses, physical and occupational therapists, and social workers were required to attend the conference, and depending on the patient’s condition, speech therapists and orthotic and prosthetic technicians could additionally participate. In the first conference, the patients’ socioeconomic characteristics, motor function, and cognitive and psychological state were shared not only with clinical experts, but also with patients, families, and caregivers, and the goals to be achieved during the hospitalization period, as well as the training and measures necessary for this, were discussed. In the second and third conferences, changes in patient’s needs and conditions were monitored, and the adequacy of the established goals and training strategies, as well as the measures set in prior conferences, were reviewed and corrected if they were deemed inappropriate. In the second or third conference, the start of the community reintegration support program other than physical and occupational therapy was decided considering the degree to which the patient perceived the need for returning home.

#### Standardization of the patient-centered multidisciplinary goal setting

Goal attainment scaling was adopted to standardize methods for goal setting and achievement evaluation [37, 38]. goal attainment scaling is a method of scoring an individualized health outcome involving goal selection and goal scaling that is standardized to calculate the extent to which a patient’s goals are met in the course of intervention [37]. Goals are individually identified to suit the patient’s needs, and each goal is rated on a 5-point scale (range − 2 to + 2) with the degree of its current and expected level of performance [37]. This approach can provide an accurate indication of success in relation to the intended goals of treatment, on the part of both the patient and the clinical experts. In the current study, we modified the original goal attainment scaling to set at least one goal for each domain of the International Classification of Functioning, Disability and Health (i.e., Body function, Participation and activities, and Environment) that the patient wants to achieve in the course of intervention to support the patient’s multidimensional need for community reintegration.

#### Multidimensionally classification of community reintegration support areas

Based on the socio-ecological model, community reintegration support is classified into the following four areas for multidimensional support: (1) area of personalized support, (2) area of family and caregiver cooperation, (3) area of multidepartmental collaboration, and (4) area of community connection. Table 2 presents the services for each area, as well as the occupational groups and expected participants involved in each service. Most of the services were designed to be multidisciplinary, with the participation of various job groups. New services were established for consulting and educating patients and caregivers to improve their capabilities, such as medical and nursing counseling, self-exercise education, welfare information provision, and education for social reintegration preparation. In addition, rehabilitation exercise, driving rehabilitation, and education for acceptance of disability, which had been operated separately from the community reintegration support team, were jointly managed by linking departments.

**Table 2:** Service configuration for each level of community reintegration service area

Area	Service title	Related occupation/dept.	Expected participants
**Area of personalized support**	Recreation	SW	PWS
	Mentoring	SW	PWS
	Psychological counseling	SW, CP	PWS
	Medical & nursing counseling	MD, NR	PWS, family/caregiver
	Self-exercise education	PT, OT	PWS, family/caregiver
**Area of family and caregiver cooperation**	Smart home experience	SW, OT	PWS, family/caregiver
	House renovation	SW, OT	PWS, family/caregiver
	Welfare information provision	SW	PWS, family/caregiver
**Area of multidepartmental collaboration**	Rehabilitation exercise	Dept. of rehabilitation exercise^a^	PWS
	Driving rehabilitation	Dept. of disability prevention and driving service^a^	PWS
	Education for acceptance of disability	Dept. of disability prevention and driving service^a^	PWS, family/caregiver
**Area of community connection**	Social life experience	SW, PT, OT	PWS, family/caregiver
	Social life training	SW, PT, OT	PWS, family/caregiver
	Vocational training	SW	PWS
	Community resources linkage	SW	PWS, family/caregiver
	Education for social reintegration preparation	SW	PWS, family/caregiver
	Community resource information provision	SW	PWS, family/caregiver

*Note*: ^a^ indicates the department responsible for designing and providing related community reintegration service in collaboration with the social reintegration support team. Unless otherwise indicated, the community reintegration support team is responsible for designing and providing services in collaboration with other departments and the patient’s community

*CP* clinical psychologist, *MD* medical doctor, *NR* nurse, *OT* occupational therapist, *PT* physical therapist, *PWS* patient with stroke, *SW* social worker

#### Development of guidelines for a tailored community reintegration support program

**Fig. 1:**
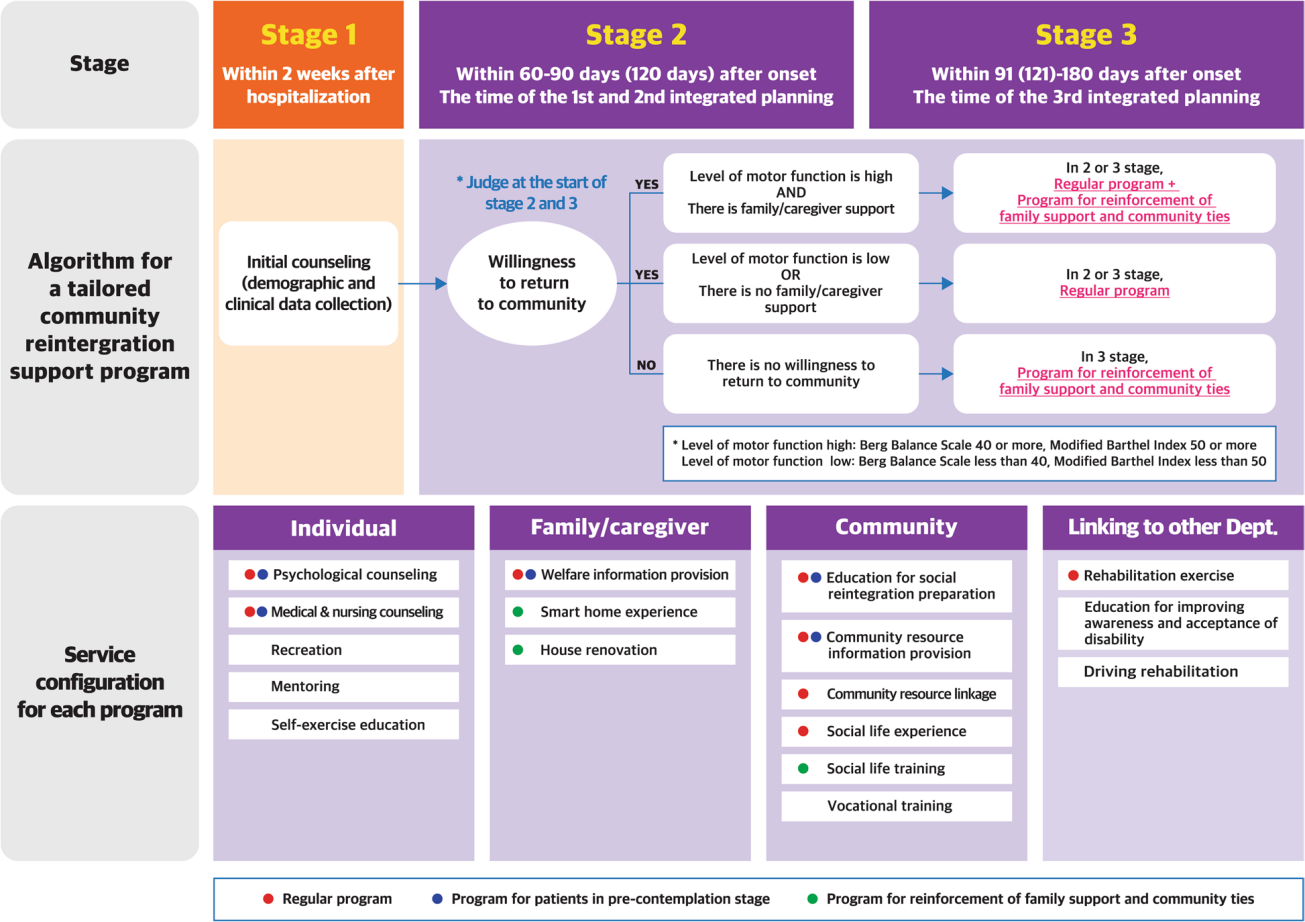
A newly developed hospital-based early supported community reintegration model

The research team developed guidelines that recommended an appropriate type of community reintegration support program according to the patient’s individual and environmental conditions. All research members agreed that the patient’s willingness to return to the community should be considered first in recommending a community reintegration support program, followed by their level of motor function and the degree of family/caregiver support. Thus, the research team classified patients into the following three groups based on those considerations and recommended a tailored program: (1) The first was a group with no willingness to return to the community. The research team judged, that these patients were still in the precontemplation stage, and recommended a community reintegration support program mainly for counseling and information provision in order to advise the patients on their exact health status and facilitate them to recognize the necessity of community reintegration. (2) The second was a group that had the willingness to return to the community but had low levels of motor function or no family/caregiver who could help patients return to their communities. The research team recommended they participate in a regular community reintegration support program but did not actively recommend services that required community connections or help from families/caregivers. (3) The third was a group that had the willingness to return to the community, a high level of motor function, and a family/caregiver who could help patients return to their communities. In this case, the research team recommended not only a regular community reintegration support program but also a program for reinforcement of family/caregiver support and community ties. Figure 1 shows the algorithm for a tailored program and the service configuration for each program.

In the algorithm for a tailored program, the level of motor function was determined using Berg Balance Scale and Modified Barthel Index scores as a result of the retrospective pilot study conducted in the course of this study. The research team sampled 95 patients discharged from the hospital between September 1, 2017, and October 31, 2018, and divided them into those who returned to the community after discharge and those who did not. In this pilot study, the research team compared the difference in the level of motor function measured using various scales between groups and found that there was a significant difference in Berg Balance Scale and Modified Barthel Index scores between 91 and 120 days after onset; when analyzing the receiver operating characteristics curve at that time, the best performance was shown in the area under the curve values at 40.0 and 49.5 points, respectively. Therefore, the research team defined Berg Balance Scale ≥40 points and Modified Barthel Index ≥50 points as reference points for recommending the program for reinforcement of family support and community ties.

## Discussion

In this study, the knowledge-to-action cycle framework was applied to develop a hospital-based early supported community reintegration model suitable for the conditions in South Korea. Through the cooperative and equitable relationship between researchers and clinical experts in the overall process of the study, we attempted to develop a more acceptable and sustainable model. Each phase of the knowledge-to-action cycle for developing the model was reported as a result of the newly developed early supported community reintegration model.

The meaning of applying knowledge translation as a research method is to find problems in the field and to find practical solutions to these problems [39]. In compliance with the knowledge translation method, this study was based on the clinical experts’ awareness of the lack of an appropriate early supported community reintegration model to operate the rehabilitation medical institution. In the process of developing the early supported community reintegration model, we recognized that the South Korean government was only now trying to introduce community care, unlike the UK and Japan, which had begun to establish community care more than a decade before South Korea. Therefore, South Korea still lacked sufficient community rehabilitation infrastructure. Taking these circumstances into account, we designed a unique hospital-based early supported community reintegration model comprehensively comprising services that would have been provided in the community after discharge in the UK and Japan, such as driving, exercise, and vocational rehabilitation, while benchmarking the best early supported discharge and Kaifukuki rehabilitation ward strategies, such as multidisciplinary goal setting and rehabilitation, as well as measures for determining the patient’s eligibility.

Another characteristic of the current study is that barriers and facilitators recognized by PWS and clinical experts were multidimensionally analyzed based on the socio-ecological model in individual, interpersonal, organizational, and community dimensions [24, 25], and community reintegration support services were also multidimensionally classified. In this study, the socio-ecological model was applied to reflect the opinion derived from the experience of clinical experts that multidimensional factors influence community reintegration of PWS. To be capable of addressing the identified multidimensional barriers and facilitators, a model was needed in which families, caregivers, and relevant organizations in the community could participate and cooperate, not just patients and clinical experts. Thus, we revised the strategies of community reintegration support-related organizations from providing hospital-based rehabilitation to providing community reintegration support in connection with hospitals and communities and reclassified the services into multiple dimensions, emphasizing the partners to cooperate and services that required cooperation.

Guidelines to provide a tailored community reintegration support program are the most notable outcome of this study. The early supported community reintegration model recommended a tailored approach of three types of community reintegration support programs according to the patient’s willingness to return to the community, level of motor function, and degree of family/caregiver support: (1) regular program, (2) program for patients in the precontemplation stage, and (3) program for reinforcement of family support and community ties. In particular, the program for patients in the precontemplation stage can borrow the idea from the transtheoretical model of behavior change [40], which assesses an individual’s readiness to act on a new, healthier behavior and provides strategies or processes of change to guide the individual. The precontemplation stage indicates the status in which people do not intend to take action in the foreseeable future. People in this stage are often unaware that their behavior is problematic or produces negative consequences, and they underestimate the benefits of changing their behavior while placing too much emphasis on the disadvantages of behavior changes. One of the most effective ways to help people at this stage is to encourage them to become more mindful of their decision-making and more conscious of the multiple benefits of changing an unhealthy behavior [40]. The transtheoretical model has been traditionally used to develop interventions and educational methods for smoking cessation, as well as weight and physical activity management [41–43]. The research team regarded patients who did not intend to return home after discharge as being in the precontemplation stage, and so to properly cope with their status, the community reintegration support program for them was composed mainly of counseling about their health and medical condition, information provision regarding welfare benefits and resources after returning to the community, and preparation for community reintegration. By contrast, the program for reinforcement of family/caregiver support and community ties is the most active type of community reintegration support program applied when the patient has both the will to return to the community and the socially supportive environment. This classification of community reintegration support programs is a tailored measure to cope with various situations faced by patients in South Korea, where the concept of community care has not yet been established and the community rehabilitation infrastructure is poor. It is expected that this guideline for a tailored community reintegration support program according to the patient’s situation can be referenced in other countries that are in a transitional stage of community care and community rehabilitation.

This study had some limitations. Since this study reported the process of developing the early supported community reintegration model through the knowledge translation method and a newly developed early supported community reintegration model, it is necessary to evaluate the effectiveness, acceptability, and sustainability of the model in future studies and to continuously supplement it for regionally optimized models. In addition, this study was conducted in a national hospital and needs to be tailored appropriately when expanded to other types of hospitals.

## Conclusions

We adopted the knowledge translation method to develop a hospital-based early supported community reintegration model that was suitable for South Korea, including the following multidisciplinary and multidimensional components in cooperation with PWS and clinical experts: revision of strategies of organizations related to community reintegration support, establishment of a multidepartmental and multidisciplinary community reintegration support system, standardization of patient-centered multidisciplinary goal setting, multidimensional classification of community reintegration support areas, and development of guidelines for a tailored community reintegration support program. In developing a guideline for a tailored community reintegration support program, we attempted to take into consideration various situations faced by PWS in South Korea to provide a transitional stage for community rehabilitation.

## Data Availability

The datasets used and/or analyzed during the current study are available from the corresponding author on reasonable request.

## Abbreviation

PWS: Patients with stroke
